# Continuous-Variable Quantum Secret Sharing Based on Thermal Terahertz Sources in Inter-Satellite Wireless Links

**DOI:** 10.3390/e23091223

**Published:** 2021-09-17

**Authors:** Chengji Liu, Changhua Zhu, Zhihui Li, Min Nie, Hong Yang, Changxing Pei

**Affiliations:** 1 State Key Laboratory of Integrated Services Networks, Xidian University, Xi’an 710071, China; liuchengjixidian@163.com (C.L.); 13381105509@189.cn (H.Y.); chxpei@xidian.edu.cn (C.P.); 2 Collaborative Innovation Center of Quantum Information of Shaanxi Province, Xidian University, Xi’an 710071, China; 3 Shaanxi Key Laboratory of Information Communication Network and Security, Xi’an University of Posts & Telecommunications, Xi’an 710121, China; niemin@xupt.edu.cn; 4 School of Mathematics and Statistics, Shannxi Normal University, Xi’an 710119, China; lizhihui@snnu.edu.cn; 5 School of Communications and Information Engineering, Xi’an University of Posts & Telecommunications, Xi’an 710121, China; 6 Institute of Spacecraft System Engineering, China Academy of Space Technology (CAST), Beijing 100094, China

**Keywords:** continuous-variable, quantum secret sharing, terahertz band, thermal state, inter-satellite communication

## Abstract

We propose a continuous-variable quantum secret sharing (CVQSS) scheme based on thermal terahertz (THz) sources in inter-satellite wireless links (THz-CVQSS). In this scheme, firstly, each player locally preforms Gaussian modulation to prepare a thermal THz state, and then couples it into a circulating spatiotemporal mode using a highly asymmetric beam splitter. At the end, the dealer measures the quadrature components of the received spatiotemporal mode through performing the heterodyne detection to share secure keys with all the players of a group. This design enables that the key can be recovered only by the whole group players’ knowledge in cooperation and neither a single player nor any subset of the players in the group can recover the key correctly. We analyze both the security and the performance of THz-CVQSS in inter-satellite links. Results show that a long-distance inter-satellite THz-CVQSS scheme with multiple players is feasible. This work will provide an effective way for building an inter-satellite quantum communication network.

## 1. Introduction

Standard point-to-point quantum key distribution (QKD) based on the fundamental laws of quantum mechanism can achieve unconditionally secure key establishment on unsafe channels [1,2,3,4]. Generally speaking, QKD can be further divided into two types according to different modulation methods, i.e., discrete-variable (DV) QKD [5,6,7,8,9] and continuous-variable (CV) QKD [10,11,12,13]. Compared with DVQKD systems, CVQKD systems can be easily integrated with traditional optical communication systems and do not necessitate single photon detectors [14,15]. At present, most QKD schemes use photons to carry encoded information through free space or telecom fiber channels for transmission. Nevertheless, as wireless communications rapidly develop, the leakage of information and the scarcity spectrum resources have become increasingly serious. Nowadays, terahertz (THz) communication is envisaged to be a key technology to meet the needs of high-speed data transmission due to the large availability of its bandwidth, especially for short-distance high-speed wireless communication [16,17] and satellite communication [18,19].

THz communication which is also conceived as one of the key technologies of 6G communication can be quantum secure by using QKD at THz bands. Compared with free-space optical communication, THz communication has the advantage of better penetrating power in the presence of fog, dust, and atmospheric turbulence, etc.; and compared with the microwave communication, it has larger capacity and better directionality. However, THz communication has a fatal weakness in the atmosphere, that is, it is easily absorbed by the pervasive atmospheric water molecules, which severely limits its communication distance [20,21]. Fortunately, THz communication is feasible in inter-satellite links since the concentration of water molecules there can be negligible and a previous work has studied the feasibility [18]. Thus, THz communication can provide an efficient path to build inter-satellite quantum communication networks.

With development of communication networks, the point-to-point QKD system may be difficult to meet the requirements of multi-party secret key sharing (at least 3 players). In this paper, we extend the standard point-to-point CVQKD to a (n,n) threshold quantum secret sharing (QSS) protocol that allows multiple players to share keys securely. The (n,n) threshold QSS which has been proven high security and efficiency with important practical applications based quantum technology [22,23,24,25], means that the dealer securely distributes the secret to *n* remote players; the secret can be recovered only by the whole group players’ knowledge in cooperation; a single player or any subset of the players in the group cannot recover the secret correctly. As a rule, QSS schemes involve more players than point-to-point QKD schemes and some players may be dishonest. Thus, QSS schemes will suffer additional attacks to make its security analysis more demanding than QKD schemes. In 2013, Lau et al. [26] utilized CVQKD technology for the first time to analyze the security of CVQSS. In 2017, Kogias et al. [27] proposed the security proof of entanglement-based CVQSS against both dishonest players and eavesdroppers appeared in the channels. Nevertheless, this protocol may hard to be implemented with the current technology when the number of players is large. Moreover, the tolerable losses of the channel in the protocol are very small.

To alleviate the implementation difficulties of entanglement-based QSS, some previous works [28,29] have proposed single qubit sequential QSS schemes. However, the general security of these schemes is still contentious [30,31]. In particular, these schemes are vulnerable to Trojan horse attacks owing to that such a design allows a spiteful eavesdropper Eve to accurately determine the corresponding polarization rotation by measuring the output of multi-photon signals she sends to the polarization rotation device of the targeted player in the link. Recently, Grice et al. [32] proposed a sequential CVQSS protocol by employing double homodyne detectors and traditional laser sources. The main idea of the protocol is that each player locally preform Gaussian modulation to prepare a standard coherent state and merges it into a circulating spatiotemporal mode by using a beam splitter. This design can prevent the eavesdropper from accessing the preparation process of quantum states, so that the protocol is immune to Trojan horse attacks. More recently, some further works [33,34,35] have been proposed to improve the performance of the sequential CVQSS protocol. Wu et al. [33] proposed a more convenient implementation of sequential CVQSS by using a thermal source and further improved the tolerance of the number of players. Liao et al. [34] further improved the maximal transmission distance of sequential CVQSS by using discrete modulated coherent states. Wang et al. [35] proposed an improved (t,n) threshold sequential CVQSS scheme based on the Lagrange interpolation formula and Gaussian modulated coherent states.

In this paper, inspired by Grice et al.’s work [32], we propose a CVQSS scheme based on thermal THz sources in inter-satellite wireless links (THz-CVQSS). In this scheme, instead of using optical photons to carry information and transmitting them by wired telecom fiber channels, we use THz photons to carry information and transmit them through wireless inter-satellite links. Similar to the original sequential CVQSS, the main idea of THz-CVQSS is that each player locally preforms Gaussian modulation to prepare a standard Gaussian-modulated thermal state (GMTS) and couples it into a circulating spatiotemporal mode using a highly asymmetric beam splitter (HABS), which can be efficiently immune to Trojan horse attacks. We apply an inter-satellite channel model to the THz-CVQSS and analyze both the security and the performance of the protocol. Simulation results strongly support the feasibility of the long-distance THz-CVQSS in inter-satellite links.

This paper is organized as follows. In Section 2, we show details of THz-CVQSS and analyze its security. In Section 3, we evaluate the secret key rate in inter-satellite links. Finally, in Section 4, we draw the conclusions.

## 2. The Proposed Quantum Secret Sharing Protocol and Its Security

**Figure 1:**
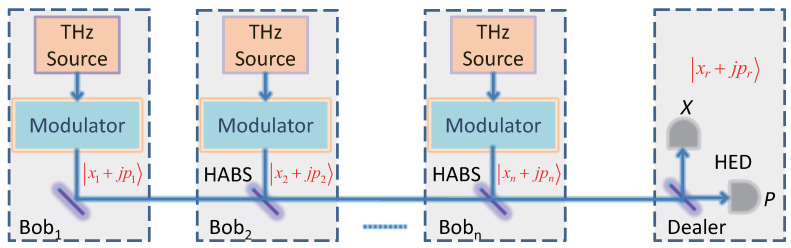
Structure diagram of THz-CVQSS system. HABS, highly asymmetric beam splitter; HED, heterodyne detector.

The schematic diagram of the proposed QSS protocol is shown in Figure 1. *n* players (Bob_1_, Bob_2_, *…*, Bob_n_) are linked with the dealer by a single quantum channel such as a inter-satellite channel (see Section 3.1 for details). For each round of quantum transmission, the first player (Bob_1_) at one far end of the link generates a pair of independent Gaussian random numbers {$x_{1},p_{1}$} (zero mean) and uses them to modulate the output of the local THz source through amplitude and phase modulators to prepare a GMTS $|x_{1}+jp_{1}\rangle$, where *j* denotes the imaginary unit. The state prepared by Bob${}_{1}$ is then sent to the adjacent player Bob${}_{2}$. For now, Bob${}_{2}$ also prepares a GMTS $|x_{2}+jp_{2}\rangle$ and couples it with the transmitted state from Bob${}_{1}$ into the same spatiotemporal mode through a HABS (the transmittance $T_{B}≅1$). All the other players in the link perform similar operations. At final, the state that the dealer receives can be expressed as $|\sum_{i=1}^{n}\sqrt{T_{i}}x_{i}+j\sum_{i=1}^{n}\sqrt{T_{i}}p_{i}\rangle$, where $T_{i}$ is the channel transmittance experienced by the quantum signal between the dealer and the *i*-th player. The dealer uses the heterodyne detector to measure quadrature components of the received states. In the case of heterodyne detection, the quantum signal is split using a balanced beam splitter. One arm is used to measure the quadrature component *X* and the other one is used to measure the quadrature component *P* after π/2 phase shift of local oscillator. This operation can allow the dealer to share a secure key which can only be recovered by all *n* players in cooperation but not by any subset of less than *n* players. The details of the protocol are as follows.

**Step 1** (Preparation). For each round of quantum transmission, *n* players (Bob${}_{1}$, Bob${}_{2}$, *…*, Bob${}_{n}$) each locally prepare a thermal Gaussian state $|x_{i}+jp_{i}\rangle \left(i=1,2,\ldots ,n\right)$ based on THz sources, Gaussian random numbers ${\left\{x_{i},p_{i}\right\}}_{i=1}^{n}$ and modulators at their stations.

**Step 2** (Transmission). First of all, the first player Bob${}_{1}$ sends the prepared state $|x_{1}+jp_{1}\rangle$ to the nearest player Bob${}_{2}$. After receiving Bob${}_{1}$’s quantum state, Bob${}_{2}$ couples his state and the received state to the same spatiotemporal mode by using the HABS. The merged quantum state is then sent to the next player.

The remaining players in the link perform similar operations, so that they can inject the locally prepared state into the same spatiotemporal mode as Bob${}_{1}$. Finally, the state arriving at the dealer’s station can be expressed as $|\sum_{i=1}^{n}\sqrt{T_{i}}x_{i}+j\sum_{i=1}^{n}\sqrt{T_{i}}p_{i}\rangle$.

**Step 3** (Detection). After receiving the quantum signal state, the dealer performs heterodyne detection to measure its quadrature components and then obtain the measurement results $\left(x_{r},p_{r}\right)$ which are kept as raw data. Repeat the above procedure many rounds until the dealer obtains sufficient raw data.

**Step 4** (Post-processing). The remaining steps use classical post-processing technologies to process these raw data.

*Step 4.1.* The dealer and all the players randomly choose and disclose a group of the raw data to deduce the channel transmittances ${\left\{T_{i}\right\}}_{i=1}^{n}$[32]. Then all the players discard their disclosed Gaussian random numbers to prevent the eavesdropper from obtaining information about the key.

*Step 4.2.* The dealer assume that all the players except Bob${}_{1}$ are dishonest (if all the players are dishonest, then the QSS protocol is meainingless).

*Step 4.3.* The dealer randomly choose another group of raw data and requests all dishonest players to disclose their corresponding Gaussian random numbers.

*Step 4.4.* The dealer can displace measurement results of the group in step 4.3 utilizing $x_{M}=x_{r}-\sum_{i=2}^{n}\sqrt{T_{i}}x_{i}$ and $p_{M}=p_{r}-\sum_{i=2}^{n}\sqrt{T_{i}}p_{i}$. Therefore, a two-party CVQKD between the dealer and Bob${}_{1}$ is established. Then they can estimate a lower bound of secret key rate (SKR) $R_{1}$ with the standard post-processing procedures in the GMTS QKD [12,36]. After that, all the players abandon the disclosed data.

*Step 4.5.* Repeat steps 4.2–4.4 *n* times. In each round, the dealer chooses a different player as the honest player and at final obtains *n* secret key rates ${\left\{R_{i}\right\}}_{i=1}^{n}$.

*Step 4.6.* Finally, the dealer employs the minimum of ${\left\{R_{i}\right\}}_{i=1}^{n}$ as the SKR of the THz-CVQSS protocol and obtains the final SKR from undisclosed data by taking advantage of the reverse reconciliation method. The dealer can use the final shared key to implement a QSS protocol. Through cooperation, *n* players can recover the shared key. However, any group of fewer *n* players cannot recover the shared key correctly, since only an exponentially small amount of information about the shared key can be obtained by them.

Next, we will analyze the security of the protocol. In a word, the proposed QSS protocol actually establishes *n* independent point-to-point CVQKD links in each round of quantum transmission. As assumed in steps 4.2–4.5, there is a honest player (Bob${}_{i}$) and the remaining n−1 dishonest players in each CVQKD. Note that this assumption is the worst case, since if all the players are dishonest, then the QSS is meaningless. In this most pessimistic case, the QSS is actually reduced to a standard CVQKD model, that is, there are two legitimate players, i.e., the sender, Bob${}_{i}$ and the receiver, Alice (the dealer). Now we need to analyze whether the remaining n−1 players (and potential eavesdroppers in the channel) can cooperate to recover the key shared by Bob${}_{i}$ and Alice. As mentioned in step 4.3, the dealer requests all the players except Bob${}_{i}$ to publish their corresponding Gaussian random numbers, which makes Bob${}_{i}$ own the complete information of all the players, while the remaining n−1 players cannot infer the information about the shared key between Bob${}_{i}$ and Alice in this CVQKD link. As a result, whether there are n−1 dishonest players or not, Alice and Bob${}_{i}$ can share a secure key. As for the eavesdroppers in the channel, we can consider their quantum attack in each individual CVQKD link. This allows us to apply the standard security proof of GMTS QKD. Some previous work [11,12] has proved the security of GMTS QKD. Thus, we can use the existing security proof for GMTS QKD to evaluate the SKR of THz-CVQSS. In addition, since the players inject the locally prepared thermal Gaussian states into the circulating spatiotemporal mode, the detection signals of the eavesdropper cannot reach the modulators within the secure stations of the honest players. In other words, the honest players can prevent the eavesdropper from accessing the preparation process of the signal states, so that the protocol is immune to Trojan horse attacks.

## 3. Secret Key Rate in Inter-Satellite Links

### 3.1. Channel Model

In recent years, THz communication is considered to be one of the key technologies to prop up the growing demand of high-speed wireless communication networks. When THz waves propagate through a free space channel, it can be impaired by turbulence, scattering, and absorption, etc. As we mentioned in the introduction, these atmosphere effects limit the transmission performance of THz waves. Nevertheless, in inter-satellite links, the beam drift effect can be neglected and the absorption of atmospheric water molecules is nearly insignificant, which can allow us to approximatively present a diffraction-only channel model as a immovable attenuation. The loss caused by the diffraction effect is only derived from the size of the diffracted beam at the receiving aperture. Thus, the transmittance *T* can be expressed as [18]

(1) $$T=1-\exp[-2{\left(r{}_{a}/l\right)}^{2}],$$

where, $r{}_{a}$ stands for the receiving aperture radius, and *l* stands for the beam radius of THz waves at transmission distance *d*. Taking advantage of the Gaussian approximation, *l* can be expressed as

(2) $$l=r_{b}\sqrt{1+{\left(\lambda d/\pi r_{b}^{2}\right)}^{2}},$$

where $r_{b}$ is the beam-waist radius, and λ is the wavelength of THz waves.

In this channel model, we assume that both the receiving aperture radius $r{}_{a}$ and the beam-waist radius $r_{b}$ to 0.6 m [13] and the environment temperature is 30 K [37].

### 3.2. Secret Key Rate

Now, we will apply inter-satellite links to estimate the SKR of the QSS protocol. According to step 4.6, the final SKR is the minimum of ${\left\{R_{i}\right\}}_{i=1}^{n}$. We assume that the transmission distance between the dealer (Alice) and the farthest player (Bob) is *d* and the other n−1 players are located between them at same intervals. Each player introduces the same amount of noise $\xi_{0}$. Theoretically, the smallest SKR comes from the farthest player. However, the smallest SKR in a realistic QSS system must be estimated according to the practical data, so it does not necessarily come from the farthest player. Thus, when Alice implement the proposed QSS protocol in practice, she should employ the experimental data to evaluate SKR of each player, and select the smallest one as the SKR of QSS. The asymptotic SKR of the protocol by using reverse reconciliation can be calculated by [38,39]

(3) $$R=\beta I_{AB}-\chi_{AE},$$

where $I_{AB}$ is the mutual information between Alice and Bob, β is the reconciliation efficiency, and $\chi_{AE}$ is the Holevo bound information available to the eavesdropper Eve and the other dishonest players on Alice’s measurement. Note that in this protocol, classical information is passed from Alice to the players for reverse reconciliation.

The transmittance of the *i*-th player in the inter-satellite channel can be calculated by

(4) $$T_{i}=1-\exp\left(-2r{{}_{a}}^{2}/l_{i}^{2}\right),$$

where,

(5) $$l_{i}=r_{b}\sqrt{1+{\left(\lambda d_{i}/\pi r_{b}^{2}\right)}^{2}},$$

and $d_{i}=\frac{n-i+1}{n}d$ is the transmission distance between the dealer and the *i*-th player. Thus, when referred to the channel input, the excess noise contributed by the *i*-th player, expressed in shot noise units, can be given by [32]

(6) $$\xi_{i}=\xi_{0}\frac{T_{i}}{T_{1}}.$$

The excess noise is an additional noise except vacuum noise which is mainly caused by the imperfection of system, e.g., modulation noise, Raman noise, background light, etc. Therefore, the total channel-added noise referred to the channel input can be defined as

(7) $$\chi_{\mathrm{line}\;}=\frac{1-T_{1}}{T_{1}}+\sum_{i=1}^{n}\xi_{i},$$

where, $\left(1-T_{1}\right)/T_{1}$ represents the channel loss. The heterodyne detection added noise referred to Alice’s input is given by

(8) $$\chi_{\mathrm{het}\;}=\frac{\left(2-\eta +2v_{el}\right)}{\eta },$$

where, η denotes the detection efficiency and $v_{el}$ denotes electronics noise of Alice’s detector.

The overall noise referred to the channel input can then be expressed as

(9) $$\chi_{\mathrm{tot}}=\chi_{\mathrm{line}}+\frac{\chi_{\mathrm{het}}}{T_{1}}.$$

The mutual information between Alice and Bob can be calculated by [12]

(10) $$I_{AB}=\log_{2}\frac{V+\chi_{\mathrm{tot}}}{1+\chi_{\mathrm{tot}}},$$

where, $V=V_{M}+V_{0}$, $V_{M}$ is the modulation variance of Bob and $V_{0}$ is the shot noise given by [12]

(11) $$V_{0}=2\bar{n}+1,$$

where,

(12) $$\bar{n}=\frac{1}{\exp\left(f\hbar /k_{T}\tau_{B}\right)-1},$$

*f* is the frequency of quantum signals, *ℏ* is Planck’s constant, $k_{T}$ is Boltzmann’s constant, and $\tau_{B}$ is the absolute temperature.

For reverse reconciliation, Eve’s information is bounded by the Holevo bound $\chi_{AE}$ which can be calculated by [39]

(13) $$\chi_{AE}=S\left(\rho_{E}\right)-\int P\left(x_{A},p_{A}\right)S\left(\rho_{E}^{x_{A},p_{A}}\right)dx_{A},p_{A}$$

where S(·) is the von Neumann entropy. $\rho_{E}^{x_{A},p_{A}}$ is Eve’s conditional density operator for the states on Alice’s measurement result. $P\left(x_{A},p_{A}\right)$ is the measured probability density. $x_{A}$ and $p_{A}$ are Alice’s measurement results. We assume that the noise and loss of Alice’s detector are trusted and cannot be accessed by Eve, then $\chi_{AE}$ can be further calculated by [39]

(14) $$\chi_{AE}=\sum_{i=1}^{2}h\left(v_{i}\right)-\sum_{i=3}^{5}h\left(v_{i}\right),$$

where $h\left(x\right)=\left(\frac{x+1}{2}\right)\log_{2}\left(\frac{x+1}{2}\right)-\left(\frac{x-1}{2}\right)\log_{2}\left(\frac{x-1}{2}\right)$, and

(15) $$v_{1,2}^{2}=\frac{1}{2}\left[\Delta_{1}\pm \sqrt{\Delta_{1}^{2}-4\Delta_{2}}\right],$$

where,

(16) $$\Delta_{1}=2T_{1}+T_{1}^{2}{\left(V+\chi_{\mathrm{line}}\right)}^{2}+V^{2}\left(1-2T_{1}\right),$$

(17) $$\Delta_{2}={\left(V\chi_{\mathrm{line}}+1\right)}^{2}T_{1}^{2},$$

(18) $$v_{3,4}^{2}=\frac{1}{2}\left[\Delta_{3}\pm \sqrt{\Delta_{3}^{2}-4\Delta_{4}}\right],$$

where,

(19) $$\begin{array}{cc} \Delta_{3}= & \frac{1}{{\left[T_{1}\left(V+\chi_{\mathrm{tot}}\right)\right]}^{2}}\left\{\Delta_{1}\chi_{\mathrm{het}}^{2}+1+\Delta_{2}+2\chi_{\mathrm{het}}\right.\\ \; & \left.\times \left[T_{1}\left(V+\chi_{\mathrm{line}}\right)+V\sqrt{\Delta_{2}}\right]+2\left(V^{2}-1\right)T_{1}\right\}, \end{array}$$

(20) $$\Delta_{4}={\left(\frac{V+\sqrt{\Delta_{2}}\chi_{\mathrm{het}}}{T_{1}\left(V+\chi_{\mathrm{tot}}\right)}\right)}^{2},$$

and

(21) $$v_{5}=1.$$

### 3.3. Simulation and Discussion

**Figure 2:**
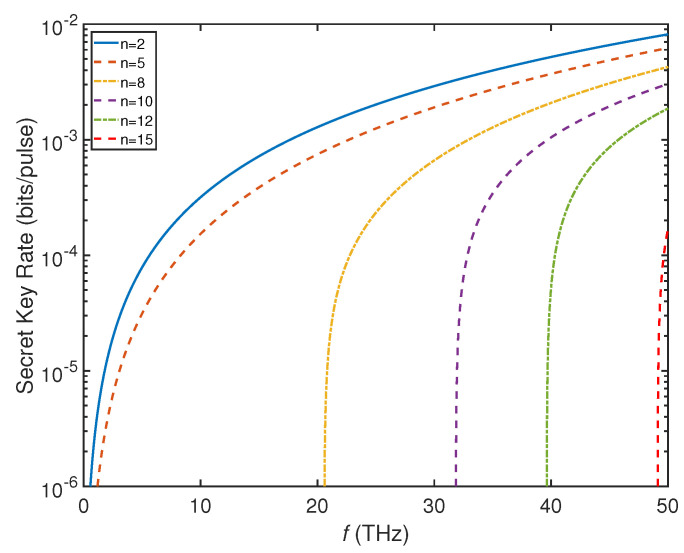
The relationship between the SKR and the frequency (0.1 THz–50 THz) with n=2,5,8,10,12,15, respectively. The simulation parameters are $\xi_{0}=0.001,\eta =0.6,v_{el}=0.1,$ β=0.98, $V_{M}=7$ and d=1500 km.

In this section, we will show the performance of THz-CVQSS. We assume that the transmission distance between the dealer and the farthest player is at least 1500 km. As shown in Figure 2, we plot the relationship between the SKR and the frequency (0.1 THz–50 THz) with n=2,5,8,10,12,15, respectively in a 1500 km inter-satellite link. We observe that as the number of players increases, the required frequency increases. In the case of the same number of players, SKR increases with higher frequency. The required frequency for a $10^{-3}$ bits/pulse SKR with n=2 is about 18 THz and a $10^{-4}$ bits/pulse SKR with n=8 is about 22 THz. When n≥5, the required frequency is at least 1 THz. When n=15, the required frequency needs to reach 49 THz. Thus, it is necessary to extend traditional THz frequency (0.1 THz–10 THz) to the mid infrared (MIR) and the far infrared (FIR) bands to study the THz-CVQSS system with high SKR and multiple players. However, in a practical communication system, the higher the frequency, the smaller the beam radius. That is to say, high-frequency communication system requires a high-precision acquisition, pointing, and tracking (APT) subsystem to communicate with the secure receiver. Here, we consider extending the frequency to a relatively suitable range (up to 50 THz) to estimate the performance of THz-CVQSS in inter-satellite links.

**Figure 3:**
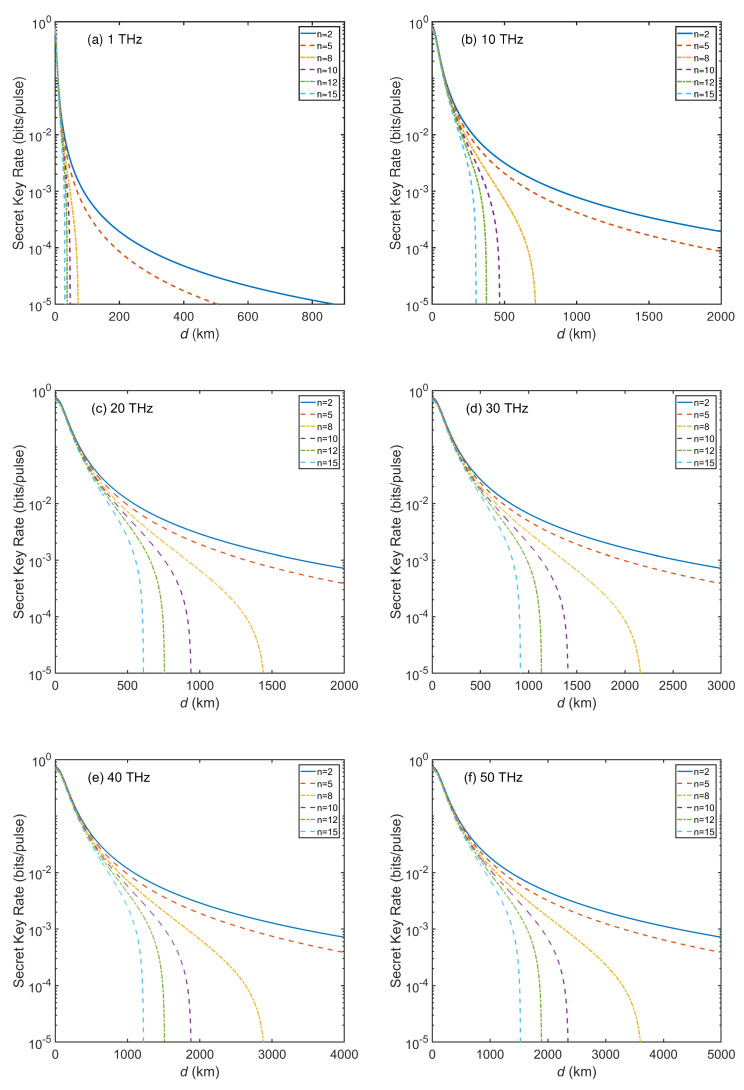
The SKR of inter-satellite THz-CVQSS versus transmission distance (km) for (**a**) f=1 THz, (**b**) f=10 THz, (**c**) f=20 THz, (**d**) f=30 THz, (**e**) f=40 THz, and (**f**) f=50 THz. The simulation parameters are $\xi_{0}=0.001,\eta =0.6,v_{el}=0.1,\beta =0.98,V_{M}=7$.

Figure 3 shows the SKR of inter-satellite THz-CVQSS versus transmission distance at different THz frequencies. We obtain that as the frequency increases, the performance of the THz-CVQSS system also improves. In Figure 3a, for n=8,f=1 THz, the maximal transmission distance can only attain 70 km. This is far from enough for long-distance communication between satellites. In Figure 3b, when the frequency is increased to 10 THz, for n=8, the maximal transmission distance can attain 715 km. This still cannot reach the assumed communication distance (1500 km). In Figure 3c, the maximal transmission distance can reach 1440 km for n=8,f=20 THz. As the number of players increases to 10 (n=10), the maximal transmission distance decreases rapidly, which can reach 940 km. In Figure 3d,e, the transmission distance can reach 1980 km and 2640 km with a $10^{-4}$ bits/pulse SKR for n=8. The results indicate that a long-distance THz-CVQSS can be achieved in the inter-satellite channel. However, when the frequency is increased to 50 THz as shown in Figure 3f, the transmission distance for n=8,10,12,15 can reach almost 3290 km, 2280 km, 1860 km and 1510 km, respectively with a $10^{-4}$ bits/pulse SKR. In particular, for n=8 and a $10^{-3}$ bits/pulse SKR, the transmission distance can exceed 1900 km. The results shown here again strongly support the feasibility of inter-satellite THz-CVQSS.

**Figure 4:**
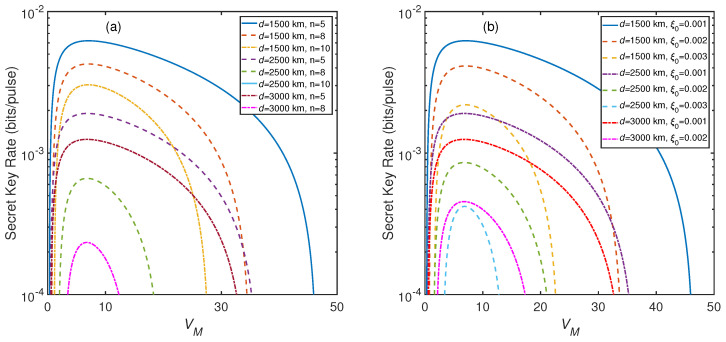
(**a**) The relationship between the SKR and the modulation variance $V_{M}$ with different transmission distance and numbers of players. $\xi_{0}=0.001,\eta =0.6,v_{el}=0.1,\beta =0.98$, and f=50 THz. (**b**) The relationship between the SKR and the modulation variance $V_{M}$ with different transmission distance and excess noise. $n=5,\eta =0.6,v_{el}=0.1,\beta =0.98$, and f=50 THz.

According to the analysis results in Figure 3, we obtain that 50 THz is an optimal frequency for our system. In order to achieve the maximal value of SKR in different scenarios, we analyze the optimal domain and optimal value of the modulation variance $V_{M}$ at 50 THz as illustrated in Figure 4. It can be clearly observed that the optimal domains of $V_{M}$ are constricted with the increase of transmission distance. In Figure 4a, as the number of players increases, the optimal domains of $V_{M}$ are also constricted. However, there is a common domain in these different optimal domains. Here, we can select the common symmetric point $V_{M}=7$ as the common optimal value. For d=3000 km, $V_{M}=7$ and n=5, the SKR can exceed $10^{-3}$ bits/pulse. We also plot the relationship between the SKR and $V_{M}$ with different excess noise $\xi_{0}$ as shown in Figure 4b. We can see that as the excess noise $\xi_{0}$ increases, the optimal domains of $V_{M}$ are constricted. Similarly, there is also a symmetric point located at $V_{M}=7$. Thus, we can obtain a common optimal $V_{M}$ as 7. In particular, we find that the SKR can achieve $2\times 10^{-3}$ bits/pulse for d=1500 km, $V_{M}=7$, and $\xi_{0}=0.003$.

**Figure 5:**
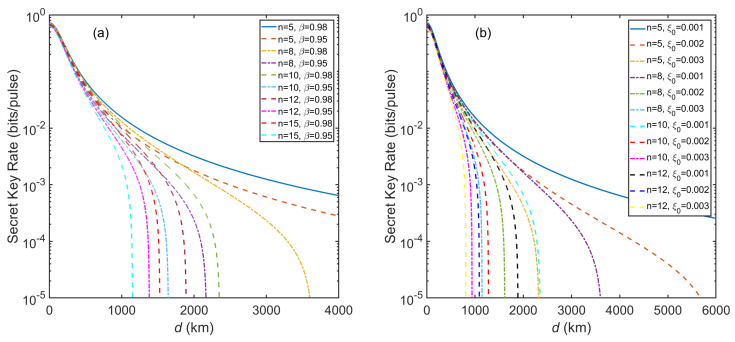
(**a**) The SKR of inter-satellite THz-CVQSS versus transmission distance (km) with different reconciliation efficiency and numbers of players. $\xi_{0}=0.001,\eta =0.6,v_{el}=0.1,V_{M}=7$, and f=50 THz. (**b**) The SKR of inter-satellite THz-CVQSS versus transmission distance (km) with different excess noise and numbers of players. $\beta =0.98,\eta =0.6,v_{el}=0.1,V_{M}=7$, and f=50 THz.

In view of Figure 5a, we analyze the influence of different reconciliation efficiency and numbers of players on the SKR with f=50 THz and $V_{M}=7$. We can observe that β is very sensitive to the influence of the maximal transmission distance. For n=8, the maximal transmission distance can reach 3600 km with β=0.98 and 2165 km with β=0.95. The difference between them is 1435 km. Nevertheless, for n=15, the difference between them is 377 km (the maximal transmission distance is 1525 km for β=0.98 and 1148 km for β=0.95). Figure 5b demonstrates the influence of different excess noise and numbers of players on the SKR. It is clear that in the case of the same number of players, $\xi_{0}$ is also very sensitive to the influence of the maximal transmission distance. For n=8, the maximal transmission distance can achieve 3600 km with $\xi_{0}=0.001$, 1615 km with $\xi_{0}=0.002$ and 1146 km with $\xi_{0}=0.003$. Interestingly, we can see that a $10^{-5}$ bits/pulse SKR at almost 2300 km can be achieved with n=10, ξ=0.001 and β=0.98 from Figure 5. This result shows the powerful performance of our THz-CVQSS system.

In short, from the aforementioned analysis, THz-CVQSS can achieve the optimal SKR with higher frequency, the optimal $V_{M}$ value, higher reconciliation efficiency and lower excess noise in inter-satellite links.

**Figure 6:**
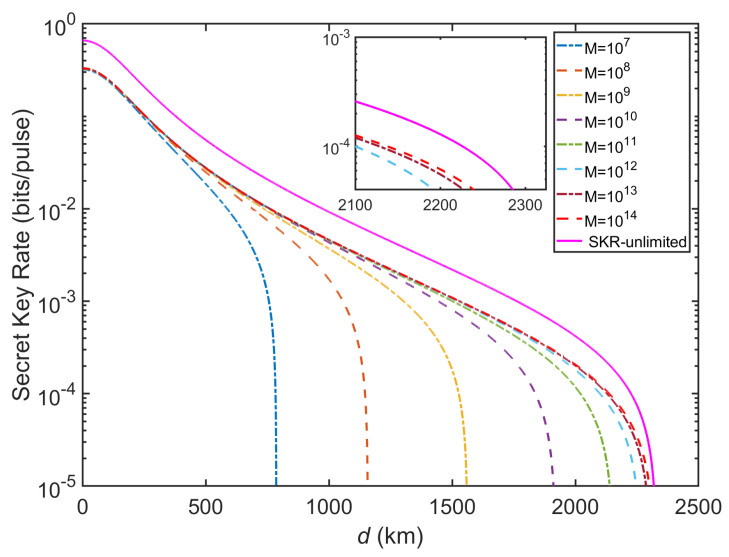
The finite-size SKR of inter-satellite THz-CVQSS versus transmission distance (km). $n=5,\xi_{0}=0.003,\eta =0.6,v_{el}=0.1,V_{M}=7$, and f=50 THz.

Due to the length of the SKR is limited at finite-size of data in the actual CVQSS system, we further consider the finite-size SKR of THz-CVQSS as shown in Figure 6. The detailed analysis of finite-size SKR is provided in Appendix A. We can observe that the maximal transmission distance increases with the increase of *M* and gradually approaches the case of SKR-unlimited. For $M=10^{14}$ and n=5, the maximal transmission distance can attain about 2300 km with ξ=0.003. Results indicate that inter-satellite THz-CVQSS can still maintain the reasonable performance in the finite-size scenario although the excess noise is comparatively high.

**Figure 7:**
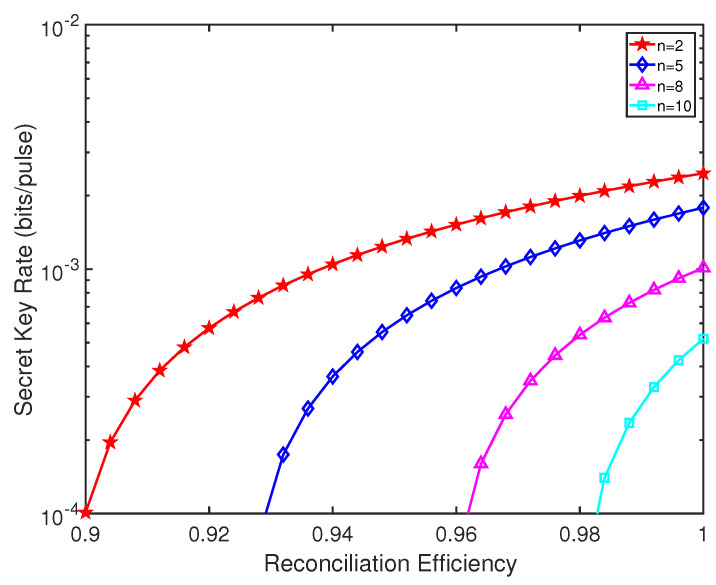
The finite-size SKR of inter-satellite THz-CVQSS versus the reconciliation efficiency with different numbers of players. $\xi_{0}=0.001,\eta =0.6,v_{el}=0.1,V_{M}=7$, d=2000 km, f=50 THz and $M=10^{10}$.

We also plot the finite-size SKR of inter-satellite THz-CVQSS versus the reconciliation efficiency with different numbers of players in view of Figure 7. In the finite-size scenario, we set the block size $M=10^{10}$ to demonstrate the available range of reconciliation efficiency. We find that as the number of players increases, the required reconciliation efficiency increases. For n=5 and d=2000 km, a $10^{-4}$ bits/pulse finite-size SKR can be achieved with the reconciliation efficiency of at least 0.93. However, when n=10, the required reconciliation efficiency exceeds 0.98. Thus, for the actual inter-satellite THz-CVQSS system, it is essential to employ an efficient reconciliation efficiency.

## 4. Conclusions

We have presented a THz-CVQSS scheme based on thermal THz sources and heterodyne detectors, which can be efficiently immune to Trojan horse attacks. On the whole, THz-CVQSS protocol actually establishes *n* independent point-to-point CVQKD links based on GMTS in each round of quantum transmission. By connecting THz-CVQSS to CVQKD based on GMTS, the security of the proposed protocol can be proved. We analyze the performance of THz-CVQSS in inter-satellite links. Results show that THz-CVQSS can achieve the optimal SKR with higher frequency, the optimal $V_{M}$ value, higher reconciliation efficiency and lower excess noise in inter-satellite links. We also verify the feasibility of inter-satellite long-distance THz-CVQSS. In particular, when the frequency is increased to 50 THz, the maximal transmission distance can reach 2300 km with the comparatively high excess noise $\left(\xi_{0}=0.003\right)$ and n=5 players in the finite-size scenario. This work can provide an effective way to build an inter-satellite quantum communication network. We expect that in future work, some non-Gaussian operations, e.g., quantum catalysis and photon subtraction, can be used to further improve the performance of THz-CVQSS.

## Appendix A. Secret Key Rate Analysis of THz-CVQSS in the Finite-Size Scenario

In the actual CVQSS system, the number of quantum signals transmitted between the dealer and the players is limited at finite-size of data, which has a profound impact on the transmission distance and SKR. Thus, in this section, we analyze the finite-size SKR of THz-CVQSS with reverse reconciliation which can be expressed by [40]

(A1) $$R_{f}=\frac{m}{M}\left(\beta I_{AB}-S_{\Theta_{\mathrm{PE}}}\left(AE\right)-\Delta \left(m\right)\right),$$

where, *m* denotes the number of quantum signals utilized to share the secure keys. *M* denotes the total number of quantum signals exchanged. h=M−m denotes the remaining number of quantum signals utilized to parameter estimation. β and $I_{AB}$ are defined in Equation (3). $S_{\Theta_{\mathrm{PE}}}\left(AE\right)$ denotes the maximal Holevo information compatible with the statistics except with probability $\Theta_{\mathrm{PE}}$ and can be calculated by the covariance matrix $\Omega_{\Theta_{\mathrm{PE}}}$. Δ(m) denotes a security parameter which is related to the privacy amplification and can be expressed by [40]

(A2) $$\Delta \left(m\right)=\frac{2}{m}\log_{2}\left(\frac{1}{\Theta_{\mathrm{PA}}}\right)+7\sqrt{\frac{\log_{2}\left(\frac{2}{\bar{\Theta }}\right)}{m}},$$

where, $\Theta_{\mathrm{PA}}$ denotes the probability of lack of success in privacy amplification. $\bar{\Theta }$ denotes the smoothing parameter.

Next, we will calculate the covariance matrix $\Omega_{\Theta_{\mathrm{PE}}}$. The finite-size SKR $R_{f}$ is minimized by $\Omega_{\Theta_{\mathrm{PE}}}$ with a probability of at least $1-\Theta_{\mathrm{PE}}$. The covariance matrix $\Omega_{\Theta_{\mathrm{PE}}}$ can be obtained by using *h* couples of correlated variables ${\left(x_{i},y_{i}\right)}_{i=1\ldots h}$. We consider a normal model for the correlated variables given by

(A3) y=qx+p,

where, $q=\sqrt{T_{1}}$ and *p* denotes a centered normal variable with the variance $\omega^{2}=T_{1}\xi_{tot}+1$, $\xi_{tot}=\sum_{i=1}^{n}\xi_{i}$. The covariance matrix $\Omega_{\Theta_{\mathrm{PE}}}$ can be calculated by

(A4) $$\Omega_{\Theta_{\mathrm{PE}}}=\left[\begin{array}{cc} VI_{2} & q_{\min}\sqrt{V^{2}-1}\sigma_{z}\\ q_{\min}\sqrt{V^{2}-1}\sigma_{z} & \left(q_{\min}^{2}\left(V-1\right)+\omega_{\max}^{2}\right)I_{2} \end{array}\right],$$

where, $q_{\min}$ is the minimal value of $\hat{q}$ and $\omega_{\max}^{2}$ is the maximal value of ${\hat{\omega }}^{2}$. The estimated value of $\hat{q}$ and ${\hat{\omega }}^{2}$ can be obtained by the maximum likelihood estimation

(A5) $$\begin{array}{cc} \hat{q} & =\frac{\sum_{i=1}^{h}x_{i}y_{i}}{\sum_{i=1}^{h}x_{i}^{2}},\\ {\hat{\omega }}^{2} & =\frac{1}{h}\sum_{i=1}^{h}{\left(y_{i}-\hat{q}x_{i}\right)}^{2}. \end{array}$$

Through the large number theorem, $\hat{q}$ and ${\hat{\omega }}^{2}$ satisfy the following distributions

(A6) $$\begin{array}{ccc} \hat{q} & ∼N\left(q,\frac{\omega^{2}}{\sum_{i=1}^{h}x_{i}^{2}}\right),\;\;\;\;\frac{h{\hat{\omega }}^{2}}{\omega^{2}} & ∼\chi^{2}\left(h-1\right). \end{array}$$

Owing to the estimated values of $\hat{q}$ and ${\hat{\omega }}^{2}$ are true values, i.e., $E\left[\hat{q}\right]=\sqrt{T_{1}}$ and $E\left[{\hat{\omega }}^{2}\right]=T_{1}\xi_{tot}+1$, we then can obtain the expressions

(A7) $$\begin{array}{cc} q_{\min} & \approx \sqrt{T_{1}}-z_{\delta }\sqrt{\frac{T_{1}\xi_{tot}+1}{hV_{M}}},\\ \omega_{\max}^{2} & \approx z_{\delta }\frac{\sqrt{2}\left(T_{1}\xi_{tot}+1\right)}{\sqrt{h}}+1+T_{1}\xi_{tot}, \end{array}$$

where, $z_{\delta }$ satisfies $1-\mathrm{erf}\left(z_{\delta /\sqrt{2}}\right)/2=\Theta_{\mathrm{PE}}/2$, here, erf(x) denotes the error function given by

(A8) $$\mathrm{erf}\left(x\right)=\int_{0}^{x}\frac{2}{\sqrt{\pi }}e^{-u^{2}}\;du.$$

Note that, we set the parameter values as

(A9) $$\begin{array}{cc} \bar{\Theta } & =\Theta_{PE}=\Theta_{PB}=10^{-10},\;\;\;\;m=h=M/2. \end{array}$$
